# Production of mixed fruit (pawpaw, banana and watermelon) wine using *Saccharomyces cerevisiae* isolated from palm wine

**DOI:** 10.1186/s40064-015-1475-8

**Published:** 2015-11-09

**Authors:** Alloysius Chibuike Ogodo, Ositadinma Chinyere Ugbogu, Amadike Eziuche Ugbogu, Chukwuma Stephen Ezeonu

**Affiliations:** Department of Microbiology, Faculty of Pure and Applied Sciences, Federal University Wukari, PMB 1020, Wukari, Taraba Nigeria; Department of Biochemistry, Faculty of Biological and Physical Sciences, Abia State University, PMB 2000, Uturu, Abia Nigeria; Department of Biochemistry, Faculty of Pure and Applied Sciences, Federal University Wukari, PMB 1020, Wukari, Taraba Nigeria

**Keywords:** Wine, *Saccharomyces cerevisiae*, Pawpaw, Watermelon, Banana

## Abstract

Pawpaw, banana and watermelon are tropical fruits with short shelf-lives under the prevailing temperatures and humid conditions in tropical countries like Nigeria. Production of wine from these fruits could help reduce the level of post-harvest loss and increase variety of wines. Pawpaw, banana and watermelon were used to produce mixed fruit wines using *Saccharomyces cerevisiae* isolated from palm wine. Exactly 609 and 406 g each of the fruits in two-mixed and three-mixed fruit fermentation respectively were crushed using laboratory blender, mixed with distilled water (1:1 w/v), and heated for 30 min with subsequent addition of sugar (0.656 kg). The fruit musts were subjected to primary (aerobic) and secondary (anaerobic) fermentation for 4 and 21 days respectively. During fermentation, aliquots were removed from the fermentation tank for analysis. During primary fermentation, consistent increases in alcohol contents (ranging from 0.0 to 15.0 %) and total acidities (ranging from 0.20 to 0.80 %) were observed with gradual decrease in specific gravities (ranging from 1.060 to 0.9800) and pH (ranging from 4.80 to 2.90). Temperature ranged from 27 °C to 29 °C. The alcoholic content of the final wines were 17.50 ± 0.02 % (pawpaw and watermelon), 16.00 ± 0.02 % (pawpaw and banana), 18.50 ± 0.02 % (banana and watermelon wine) and 18.00 ± 0.02 % (pawpaw, banana and watermelon). The alcoholic content of the wines did not differ significantly (p > 0.05). The pH of all the wines were acidic and ranged from 2.5 ± 0.01 to 3.8 ± 0.01 (p > 0.05). The acid concentration (residual and volatile acidity) were within the acceptable limit and ranged from 0.35 ± 0.02 to 0.88 ± 0.01 % (p > 0.05). Sensory evaluation (P > 0.05) rated the wines acceptability as ‘pawpaw and banana wine’ > ‘pawpaw and watermelon’ > ‘pawpaw, watermelon and banana’ > ‘banana and watermelon wine’. This study has shown that acceptable mixed fruit wines could be produced from the fruits with *S. cerevisiae* from palm wine.

## Background

Fruit juices are fermented to produce wine, an alcoholic beverage. Grapes are usually preferred because of the natural chemical balance of the grape juice which aids their fermentation process without the addition of sugars, acids, enzymes, or other nutrients. However, fruits such as banana, cucumber, pineapple and other fruits are used in wine production (Obaedo and Ikenebomeh 2009; Chilaka et al. 2010; Noll 2008).

Home-made wine production has been practiced with various fruits such as apple, pear and strawberry, cherries, plum, banana, pineapple, oranges, cucumber, watermelon, guava, etc. Using species of *Saccharomyces cerevisiae* which converts the sugar in the fruit juices into alcohol and organic acids, that later react to form aldehydes, esters and other chemical compounds which also help to preserve the wine (Fleet 2003; Duarte et al. 2010; Isitua and Ibeh 2010). Yeasts from other sources such as palm wine has also been used (Ayogu 1999) in the production of fruit wine.

Banana (*Musaacuminata*) is an important staple starchy food in Nigeria. Ripe bananas are consumed raw as a desert fruit. Banana serves as good nutritional sources of carbohydrates, minerals such as potassium and vitamins such as B_1_, B_2_, B_3_, B_12_, C and E. Following the high nutritional content of banana, it is consumed in large quantity in a variety of ways in Africa. The banana fruit can be eaten raw or cooked (e.g. deep fried, dehydrated, baked in its skin or steamed), processed into flour or fermented for the production of beverages such as banana juice, beer (e.g. *mbege* brewed by the Chagga people in the Kilimanjaro region of Tanzania), vinegar and wine (Pillay et al. 2004; Nelson et al. 2006; Pillay and Tripathi 2007). However, banana has a short shelf-life under the prevailing temperature and humidity condition in tropical countries, including Nigeria. This results to wastage of the fruits as a result of poor handling and inadequate storage facilities (Akubor et al. 2003; Wall 2006). Moreover, fermenting banana juice into wine is considered to be an attractive means of utilizing surplus banana, since the consumption of banana wine provides a rich source of vitamins and ensures harnessing of the fruits into a useful by-product (Obaedo and Ikenebomeh 2009).

Pawpaw (*Caricapapaya*) is grown mostly for fresh consumption or for production of latex. *C.papaya* plants produce natural compounds (annonaceous, acetogenins) in leaf bark and twig tissues that possess both highly anti-tumour and pesticidal properties (Nwofia and Ojimelukwe 2012; Nwofia and Okwu 2012). The papaya fruit, as well as all other parts of the plant, contain a milky juice in which an active principle known as papain is present which has value as a remedy in dyspepsia and has been utilized for the clarification of beer. The juice has been in use on meat to make it tender, (Ayoola and Adeyeye 2010). The unripe fruit is used as a remedy for ulcer and impotence. It cleans bacteria from the intestines and hence encourages the absorption of vitamins and minerals, especially vitamin B_12_. The tea prepared with the green papaya leaf, promotes digestion and aids in the treatment of ailments such as chronic indigestion, overweight and obesity, arteriosclerosis, high blood pressure and weakening of the heart (Nwofia and Ojimelukwe 2012). However, ripe pawpaw fruits are very perishable, and large quantities are disposed off yearly due to lack of or poor storage facilities resulting to loss of the vital nutrients contained in the pawpaw fruits (Awe 2011; Souza et al. 2008; Nwofia and Okwu 2012; OECD 2010; Ugbogu and Ogodo 2015). However, these losses can be reduced and pawpaw can be made available all year round, by utilizing the fruits for other purposes such as wine production.

Watermelon (*Citrullusvulgaris* L.) is a tropical fruit which grows in almost all parts of Africa and South East Asia (Koocheki et al. 2007). It serves as a good source of vitamins and phytochemicals that have chemopreventive effects against cancer Perkins-Veazie and Collins 2004; Collins et al. 2005; Oms-Oliu et al. 2009; Enukainure et al. 2010; Inuwa et al. 2011). In Nigeria, watermelon are fermented, blended and consumed as juice, nectars, fruit cocktails and can also be used as an appetizer or snacks, depending on how it is prepared (Kerje and Grum 2003; Onyeleke and Olaniyan 2007; Oms-Oliu et al. 2009; Enukainure et al. 2010). The seeds are also reported to possess medicinal properties and are used to treat chronic or acute eczema. It contains high levels of proteins, lipids and is a rich source of carbohydrate and fibre. Arginine, glutamic acid, aspartic acid and leucine are the predominant amino acids in watermelon proteins. Reports are also available on the biological value, true digestibility, protein efficiency ratio and net protein utilisation of watermelon seeds (Wani et al. 2011; Lawal 2011; Inuwa et al. 2011). Moreover, they are used as a domestic remedy for urinary tract infection, hepatic congestion, catarrh, worm remedy, abnormal blood pressure (Amadi et al. 2003). Watermelon contain large amount of beta carotene and are significant sources of lycopene (Collins et al. 2005). The production of wine from common fruits could help reduce the level of post-harvest losses and increases the variety of wines (Okoro 2007; Alobo and Offonry 2009).

Palm wine is a refreshing alcoholic beverage widely consumed in southern Nigeria, Asia and southern America (Elijah et al. 2010). It is obtained from the sap of palm trees such as oil palm (*Elaeisguiniensis*) and *Raphia* palm (*RaphiaHookeris* and *R*. *vinifera*) (Okafor 2007). Palm wine is presented in a variety of flavours, ranging from sweet (unfermented) to sour (fermented) and vinegary. It is produced by a succession of microorganisms, Gram-negative bacteria, lactic acid bacteria and yeasts as well as acetic acid bacteria. Yeasts isolated from palm wine have been identified as coming from various genera such as *Saccharomyces*, *Pichia*, *Schizosaccharomyces,Kloekera*, *Endomycopsis*, *Saccharomyeoides* and *Candida* which find their way into the wine from a variety of sources including air, tapping utensils, previous brew and the trees. Hence, palm wine serves as a source of single cell protein and vitamins (Fleet 2003; Ezereonye 2004; Okafor 2007; Duarte et al. 2010; Adedayo and Ajiboye 2011). The major fermentation is undertaken by about twenty indigenous strains of *S. Cerevisiae* which are genetically different from the strains used to make wine from grapes and have the capability to survive and continue fermentation process up to ethanol concentration of 18 %, making them ideal for producing ethanol (Ezeogu and Emeruwa 1993; Legras et al. 2007; Noll 2008).

Though, studies have shown that bananas, pawpaw and watermelon (Obaedo and Ikenebomeh 2009; Enukainure et al. 2010; Awe 2011) and several other fruits including, pineapple (Isitua and Ibeh 2010), carrot Monsavi et al. 2011), mango (Reddy and Reddy 2005), guava (Kocher and Pooja 2011) can be used in wine production, the combination of these fruits in wine production is not readily available in literature. This paper reports the production and the quality of wine made from mixed fruits of banana, pawpaw and watermelon using *S*. *cerevisiae* isolated from palm wine.

## Methods

### Source of materials

Mature ripe banana (*M. acuminata*), pawpaw (*C. papaya L*), and watermelon (*C. vulgaris L.*) were purchased from the local central market (Nkwo Achara) in Uturu, Abia State, Nigeria. Fresh palm wine from *Raphia hookeri* were obtained from the palm wine tappers in Uturu within 1 h of tapping. The fruits and the palm wine were transported to the laboratory in clean cellophane bags and in an ice box respectively for analysis.

### Isolation of *S. cerevisiae* from palm wine

Culturing of the fresh palm wine was done on Potato Dextrose Agar (PDA) and incubated at room temperature for 24 h. Nineteen isolates were obtained and sub-cultured on fresh medium to obtain pure cultures. The yeast cultures were transferred to modified Malt Extract Agar (MEA) containing yeast extract and 2 % glucose and then incubated for 24 h. Out of the 19 isolates, six were identified as *S. cerevisiae* based on their cultural characteristics, microscopy and their pattern of fermentation and assimilation of glucose, sucrose, raffinose, galactose, maltose, dextrose, trehalose and meliobiose as described by Amoa-Awua et al. (2006). The different isolates of *S. cerevisiae* were further screened for their ability to tolerate different concentrations of sugar and alcohol by inoculating on MEA supplemented with 10–60, and 5–30 %, sucrose and ethanol respectively. The isolate with the highest sugar and alcohol tolerance was selected and used as the starter culture. The identified organism was maintained on MEA slant.

### Multiplication of starter culture

The isolated organism was multiplied prior to fermentation by culturing them on Malt Extract Broth (MEB) and incubating for 48–72 h at 27.0 °C ± 0.02. The broth cultures of the organism were centrifuged at 500 rpm for 5 min. The sediments were collected and used for must fermentation.

### Preparation of must for mixed fruit fermentation

The must was prepared for two-mixed fruit and three-mixed fruit fermentation respectively. The fruits were washed thoroughly with distilled water and then peeled. Exactly 609 and 406 g each of the fruit samples, banana, pawpaw and watermelon were weighed for two-mixed fruit and three-mixed fruit fermentations respectively. This was then chopped into smaller pieces using a clean knife before transferring them quantitatively into laboratory blender for crushing. The crushed sample was transferred into a clean new transparent bucket and mixed with distilled water (1:1 w/v). Exactly 0.656 kg of sugar was added to the must followed by vigorous stirring. Exactly 4 g of sodium metabisulphate (Na_2_S_2_O_5_) was dissolved in 400 ml of water and poured in 100 ml aliquots to each of the mixtures and stirred properly. Sodium metabisulphate serve as a sterilizer and prevents fermentation before the addition of the yeast starter. The sugar concentrations were measured and the musts were mixed in the combination of ‘pawpaw and watermelon’ (30.4 °Brix), ‘pawpaw and banana’ (29.3 °Brix), ‘pawpaw, banana and watermelon’ (32.1 °Brix) and then ‘banana and watermelon’ (31.2 °Brix).

### Preparation of yeast starter culture

The yeast starter culture was prepared from a known quantity of the must for fermentation, small quantity of sugar, yeast and a known volume of water. The mixture of all these were treated with yeast nutrients and allowed to stand for 24 h. Approximately 200 ml of water was boiled and allowed to attain 37 °C and 200 ml of each mixture of the must (banana and pawpaw, banana and watermelon, pawpaw and watermelon, and banana, pawpaw and watermelon) respectively treated with sugar was added. Exactly 5 g of citric acid was added to each of the preparations and then stirred for proper mixing. Exactly 2 g each of the yeast nutrient namely Potassium phosphate, Ammonium sulphate and Magnesium sulphate was dissolved in 100 ml of distilled water and poured to each of must mixture. Exactly 3.7 ml representing approximately 10^8^ cfu/ml (measured using McFarland standard) of the yeast (*S. cerevisiae*) isolated from palm wine after centrifugation was added to each of the mixture, stirred properly and allowed to stand for 24 h before use.

### Fermentation

The primary fermentation was initiated by the addition of the starter culture. The must was stirred every 12 h with subsequent reading of the specific gravity, pH, temperature and alcohol content for 4 days. After 4 days, the wine was racked into the secondary fermenter. The secondary fermentation was done in an air tight container in which a tube was passed into a clean bottle containing clean water. The essence was to monitor the course of fermentation. This was allowed until completion of fermentation as was evidenced by lack of the appearance of bubbles in the container usually within 3 weeks. Secondary fermentation was done for 21 days. When fermentation stopped, the wine was promptly racked off the lees ensuring minimum exposure to oxygen. After secondary fermentation, the wines were clarified. The clarification/fining were done using bentonite (a clarifying agent). Exactly 500 g of bentonite was dissolved in two litres of boiling water and stirred properly to a gel form. This was allowed to stand for 24 h. Then 150 g of the gel-like bentonite was transferred into each of the wine followed by stirring to dissolve properly. A small quantity of the mixture was collected in a clean bottle which was covered tightly and was used to monitor the process of clarification. This was done for a period of 3 months. Filtration was done after the wines had completed clarification using muslin cloth, sieve and syphon tubes sterilized by 70 % alcohol. The wines was syphoned into the sieve containing four layers of muslin cloth. The residues were removed and the filtrates were allowed to mature for a period of 6 months before other chemical analysis was carried out.

### Isolation of microorganisms from the fermentation broth

Microbial analysis of the fermentation broth was performed as described by Fleet (2003) using Nutrient Agar (NA), MacConkey Agar (MA) and Potato Dextrose Agar (PDA). The nutrient agar used was treated with fulcin (50 mg/20 ml of NA) to suppress fungal growth while the PDA was treated with chloramphenicol. The cultured plates were incubated at room temperature and pure cultures were obtained by streaking and identified based on colonial characteristics, microscopy, biochemical reactions and carbohydrate utilization (Fawole and Oso 1988; Onyeagba 2004). The fungi were identified only on the basis of their cultural characteristics and microscopy (Isitua and Ibeh 2010 Barnett et al. 2000).

### Chemical analysis of the wines

The volatile acidity was determined using the method described by McClements (2003), total acidity of the wines was determined by titration and concentration of the acid was calculated. The residual acidity of the wines was also determined as described by McClements (2003) while the alcohol content was determined using the density method. The specific gravities of the wines were determined using the hydrometer method and the results were determined from the reading on the stem (Awe 2011). The total solid and total sugar content of the wines were determined using the method of McClements (2003) and the pH and temperature were determined using a digital pH metre and an analytical thermometer respectively.

### Sensory evaluation

The wines produced were compared for colour, flavour, taste, clarity, and overall acceptability by a panel of twenty judges on a seven point hedonic scale where seven denotes excellent and one very poor.

### Statistical analysis

The completely randomized analysis of variance (ANOVA) was used as described by (Winner 2004) to analyze the data obtained. Mean separation and comparison was done using SPSS version 16.0. Significance was accepted at P < 0.05 and results were expressed as mean ± standard deviation from the mean.

## Results

The morphological and physiological characteristics of the yeast isolated from palm wine are represented in Table 1.

**Table 1:** Characteristics of *S. cerevisiae* isolated from palm wine

Colony features	Microscopy	Gram reaction	Ascospore	Growth at 37 °C on SDA	Germ tube	KNO_3_	Glucose	Dextrose	Maltose	Sucrose	Galactose	Raffinose	Trehalose	Lactose	Mannitol	Melibiose	Cellobiose	Xylose	Dulcitol
Smooth, moist, white to cream coloured colonies	Spherical, elongated cells with multilateral budding	Positive, ascospore negative	+	+	−	−	+	+	+	+	+	+	+	−	−	−	−	−	−

+, positive; −, Negative

**Fig. 1:**
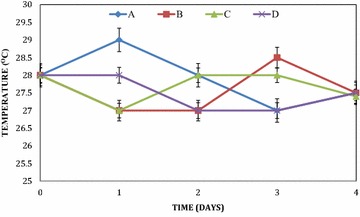
Temperature variations of the wines during primary fermentation. *A* Pawpaw and watermelon wine, *B* pawpaw and banana wine, *C* pawpaw, banana and watermelon wine, *D* banana and watermelon wine

**Fig. 2:**
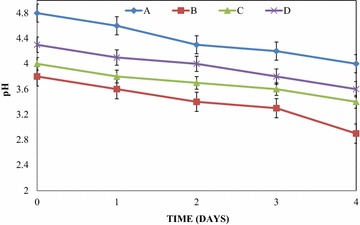
pH variations of the wines during primary fermentation. *A* pawpaw and watermelon wine, *B* pawpaw and banana wine, *C* pawpaw, banana and watermelon wine, *D* banana and watermelon wine

There were fluctuations in the temperature of the mixed fruit wines throughout the period of fermentation (Fig. 1). These variations were observed for all the wines. In all the mixed fruit wines, the temperatures were observed to range from 27.0 °C ± 0.02 to 29.0 °C ± 0.02. The pH in the mixed fruit wines was acidic throughout the period of fermentation. This was also irrespective of the fruit wine. The pH ranged from 4.0 ± 0.01 to 4.8 ± 0.01 in pawpaw and watermelon wine, 2.9 ± 0.01 to 3.8 ± 0.01 in pawpaw and banana wine, 3.4 ± 0.01 to 4.0 ± 0.01 in pawpaw, banana and watermelon wine and 3.6 ± 0.01 to 4.3 ± 0.01 in banana and watermelon wine (Fig. 2).

**Fig. 3:**
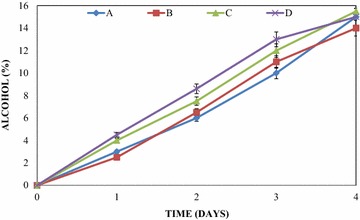
Alcohol content variations of the wines during primary fermentation. *A* pawpaw and watermelon wine, *B* pawpaw and banana wine, *C* pawpaw, banana and watermelon wine, *D* banana and watermelon wine

A steady increase in alcohol content was observed in the mixed fruit wines throughout the period of primary fermentation (Fig. 3). This increase was observed in all the mixed fruit wines irrespective of the fruits used. The concentration of alcohol in the mixed fruit wines at the end of primary fermentation were observed to range from 0 to 15, 0 to 14, 0 to 15.5 and 0 to 15 % in pawpaw and watermelon wine, pawpaw and banana wine, pawpaw, banana and watermelon wine and banana and watermelon wine respectively. The highest alcohol content was observed in the wine produced by the mixture of pawpaw, banana and watermelon (15.5 %), while the least alcohol content was observed in pawpaw and banana wine (14 %). The specific gravities of the mixed fruit wines gradually decreased throughout the period of primary fermentation. After primary fermentation, specific gravity values were observed to range from 0.9800 to 1.0600, from 0.9820 to 1.0300, from 0.9800 to 1.030 and from 0.9810 to 1.0900 in pawpaw and watermelon wine, pawpaw and banana wine, the three mixed fruit wines and banana and watermelon wine respectively.

**Fig. 4:**
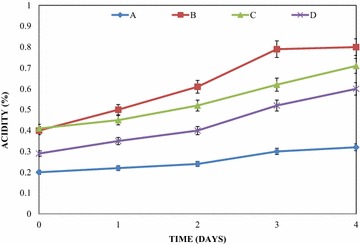
Variations in the total acidity of the wines during primary fermentation. *A* pawpaw and watermelon wine, *B* pawpaw and banana wine, *C* pawpaw, banana and watermelon wine, *D* banana and watermelon wine

Figure 4 showed the trend in total acid concentrations in the mixed fruit wines during the primary fermentation period with the test yeast. As shown in the figures, total acidity was observed to show steady increase with time throughout the period of primary fermentation. These increases were irrespective of the test fruit wine. At the end of primary fermentation, acid concentration in the pawpaw and watermelon wine was observed to increase from initial concentration of 0.20 ± 0.01 to final concentration of 0.32 ± 0.02 %. Similarly, total acidity was observed to increase from initial concentration of 0.40 ± 0.02 to a final concentration of 0.80 ± 0.02 %, 0.41 ± 0.01 to 0.71 ± 0.01 % and 0.29 ± 0.02 to 0.62 ± 0.01 % for pawpaw and banana wine, the three mixed fruit wine and banana and watermelon wine respectively.

After secondary fermentation, the temperature of the wines were observed to range from 27 ± 0.07 °C for pawpaw and banana wine to 28 ± 0.07 °C for pawpaw, banana and watermelon wine and banana and watermelon wine. The pH of the wines maintained an acidic range of 2.7 ± 0.1 for pawpaw and banana wine to 3.9 ± 0.1 for pawpaw and watermelon wine. There were little increases in the alcoholic content of the mixed fruit wines after secondary fermentation. The alcohol content of pawpaw and watermelon wine increased from 15 ± 0.02 % in primary fermentation to 16.5 ± 0.02 % after secondary fermentation, 14 ± 0.02 to 15.2 ± 0.02 %, 15.5 ± 0.02 to 17.5 ± 0.02 % and 15 ± 0.02 to 18 ± 0.02 % for pawpaw and banana wine, pawpaw, banana and watermelon wine and banana and watermelon wine respectively. The highest alcohol content was observed in banana and watermelon wine (18 ± 0.02 %) while pawpaw and banana wine recorded the lowest alcohol content (15.2 ± 0.02 %). In the case of specific gravities, little decreases were also observed in all the wines after secondary fermentation with banana and watermelon wine having the lowest value (0.9770 ± 0.00) and, pawpaw and banana wine having the highest value (0.9800 ± 0.00) while the acid concentrations ranged from 0.34 ± 0.02 (pawpaw and watermelon wine) to 0.86 ± 0.02 % (pawpaw and banana wine) (Table 2).

**Table 2:** Temperature, pH, specific gravity, alcohol content and total acidity of the wines after secondary fermentation

Wines	Temp (^o^C)	pH	Specific gravity	Alcohol (%)	Total acidity (%)
A	28.00 ± 0.7	3.90 ± 0.1	0.9790 ± 0.00	16.50 ± 0.2	0.34 ± 0.02
B	27.00 ± 0.7	2.70 ± 0.1	0.9800 ± 0.00	15.20 ± 0.2	0.86 ± 0.01
C	27.50 ± 0.7	3.10 ± 0.1	0.9780 ± 0.00	17.50 ± 0.2	0.74 ± 0.01
D	28.00 ± 0.7	3.20 ± 0.1	0.9770 ± 0.00	18.00 ± 0.2	0.63 ± 0.02

Values are expressed as mean ± standard deviation

*A* pawpaw and watermelon wine, *B* pawpaw and banana wine, *C* pawpaw, banana and watermelon wine, *D* banana and watermelon wine, *%* percentage, *Temp* Temperature

The general chemical parameters of the mixed fruit wines after maturation compared favourably. The result indicated that the final alcohol concentration of pawpaw and watermelon wine, pawpaw and banana wine, pawpaw, banana and watermelon wine, and banana and watermelon wine, were 17.50 ± 0.02, 16.00 ± 0.02, 18.00 ± 0.02 and 18.50 ± 0.02 % respectively (Table 3). These variations do not show any significant difference (p > 0.05).

**Table 3:** Chemical parameters of the final wines

Chemical parameters	Wines				*P* value
	A	B	C	D	
Alcohol content (%)	17.50 ± 0.02	16.00 ± 0.02	18.00 ± 0.02	18.50 ± 0.02	>0.05
Total acidity (%)	0.35 ± 0.02	0.88 ± 0.01	0.78 ± 0.02	0.65 ± 0.01	>0.05
Residual acidity (%)	0.13 ± 0.02	0.30 ± 0.02	0.28 ± 0.02	0.22 ± 0.02	>0.05
Volatile acidity (%)	0.24 ± 0.02	0.58 ± 0.02	0.48 ± 0.02	0.44 ± 0.02	>0.05
Specific gravity (kg/l)	0.9784 ± 0.00	0.9800 ± 0.00	0.9780 ± 0.00	0.9740 ± 0.00	>0.05
Density (kg/l)	0.9840 ± 0.00	0.9880 ± 0.00	0.9880 ± 0.00	0.9880 ± 0.00	>0.05
Total solids (%)	0.16 ± 0.02	0.55 ± 0.02	0.43 ± 0.02	0.29 ± 0.02	>0.05
Total sugar (%)	0.77 ± 0.02	0.94 ± 0.02	0.64 ± 0.02	0.54 ± 0.02	>0.05
pH	3.80 ± 0.01	2.50 ± 0.01	3.00 ± 0.01	3.40 ± 0.01	>0.05
Temperature (^o^C)	28.00 ± 0.07	27.50 ± 0.07	28.00 ± 0.07	27.00 ± 0.07	>0.05

Values are expressed as mean ± standard deviation; Significant different are taken at P < 0.05

*A* pawpaw and watermelon wine, *B* pawpaw and banana wine, *C* pawpaw, banana and watermelon wine, *D* banana and watermelon wine

Sensory evaluation (p > 0.05) rated the acceptability of the wines as pawpaw and banana wine > pawpaw and watermelon > pawpaw, watermelon and banana > banana and watermelon wine (Table 4).

**Table 4:** Sensory evaluation of the mixed fruit wines

Parameters	Wines				P value
	A	B	C	D	
Taste	4.4	4.8	4.1	3.7	>0.05
Clarity	4.7	4.7	4.5	3.6	>0.05
Colour	4.5	5.0	4.7	3.9	>0.05
Flavour	4.7	4.6	4.3	3.8	>0.05
Overall acceptability	4.6	4.9	4.6	4.1	>0.05

The wine colours are pale yellow (pawpaw and watermelon wine), straw yellow (pawpaw and banana wine), dark brown (pawpaw, banana and watermelon wine) and cream colour (banana and watermelon wine)

*A* pawpaw and watermelon wine, *B* pawpaw and banana wine, *C* pawpaw, banana and watermelon wine, *D* banana and watermelon wine

## Discussion

The fermentation of wine is known to be complex with various ecological and biochemical processes involving yeast strains (Fleet 2003). The fermentation for the elaboration of beverage is known to depend on the performance of the yeast to convert the sugars into alcohol and esters. Besides, the different species of yeast that develop during fermentation determine the characteristics flavour and aroma of the final product (Duarte et al. 2010). Also, because different fruits have different composition, there is the need for yeast strains to adapt to different environments, such as sugar composition and concentration of acetic acid (Fleet 2003; Chilaka et al. 2010; Duarte et al. 2010).

The mixed fruit wines (pawpaw and watermelon wine, pawpaw and banana wine, pawpaw, banana and watermelon wine and banana and watermelon wine) produced in the present investigation revealed low pH values (in the range of 2.5–3.8) throughout the fermentation periods and in the final product. Similar observations have been reported for other tropical fruit wines such as tundu wine (Sahu et al. 2012), sweet potato wine (Ray et al. 2011), sapota fruit wine (Panda et al. 2014a, b) and banana wine (Obaedo and Ikenebomeh 2009). Studies have shown that during fermentation of fruit, low pH is inhibitory to spoilage organisms but increases conducive environment for the growth of desirable organisms. Also, low pH is known to give fermenting yeasts a competitive advantage in natural environment (Reddy and Reddy 2005; Chilaka et al. 2010). The decrease in pH could be due to accumulation of organic acids during fermentation and this reduces the influence of bacteria that can lead to spoilage. Therefore the wines have a good keeping quality.

Fluctuations in temperature of the must were observed during the period of fermentation. This could be as a result of biochemical changes occurring during the metabolism of the substrates by the fermenting organism. Temperature of the final mixed fruit wines ranged from 27.00 ± 0.07 to 28 ± 0.07 °C.

The present study also revealed a consistent increase in the total acidity of the mixed fruit wines throughout the period of fermentation. The total acidity of final wine is expected to be between 0.5 and 1.0 % (Chilaka et al. 2010). In this study, the result of the total acidity in the mixed fruit wines fell within this limit ranging from 0.35 ± 0.02 to 0.88 ± 0.01 %. However, the acidity is lower than the reports of Ray et al. (2011) for sweet potato wine (1.34 g/100 ml) and Panda et al. (2014a) for sapota fruit wine (1.29 g/100 ml) but is consistent with the report of Panda et al. (2014b) who reported 0.15 ± 0.07 g/100 ml for bael wine. High acidity is known to favour the fermentative and competitive advantage of yeasts in natural environment as reported by Reddy and Reddy (2005). This acidity was observed to be more of volatile acidity than the residual acidity. This implies that even if the wines are consumed in large quantities, the acidity level can easily be removed by the body system. Moreover the acidity (volatile and residual) of the wines in the present study do not differ significantly (p > 0.05).

In order to supplement the sugar content of the musts, sucrose was part of the additives. Reports have shown that the major problem associated with the use of tropical fruits in wine production is their low sugar contents (Alobo and Offonry 2009). In the present study, the fermentation was nearly complete with total sugar content of 0.76 ± 0.02, 0.94 ± 0.02, 0.64 ± 0.02, and 0.54 ± 0.02 % in ‘pawpaw and watermelon wine’, pawpaw and banana wine’, ‘pawpaw, banana and watermelon wine’ and banana and watermelon wine respectively. This observation did not correspond with the reports of Panda et al. (2014a), Ray et al. (2011), Sahu et al. (2012) and Panda et al. (2014b) who reported higher values for sapota fruit wine (3.28 g/100 ml), purple sweet potato wine (1.35 g/100 ml), tendu wine (3.78 g/100 ml) and bael wine (2.05 ± 0.12 g/100 ml) respectively. The result revealed that the total sugar contents of the wines in the present study are less than 1 %. This is an indication that the wines will have a good keeping quality since the fear of further fermentation during storage which could lead to spoilage will not arise. This result also showed that the wines could be classified as dry table wines because of low total sugar content of less than 1 %. The variations in the total sugar content of the wines were not observed to differ significantly (p > 0.05).

The total solids obtained in the wines were low ranging from 0.16 ± 0.02 % to 0.46 ± 0.02 in pawpaw and watermelon wine and pawpaw and banana wine respectively, and do not differ significantly (p > 0.05). This could be attributed to the efficiency of the yeast in fermentation. It also implies that consumers are not exposed to the risk of taking in too much solid into the body. However, reduction in the total solid of the wines could be achieved by further filtrations.

Remarkable amount of alcohol were produced from the fruit wines during fermentation with the test yeast (*S.cerevisiae* from palm wine). This trend was consistent in all the wines. In general, the percentage alcohol produced from the respective mixed fruit wines at the end of fermentation by the test yeast were 17.50 ± 0.02 % (pawpaw and watermelon wine), 16.00 ± 0.02 % (pawpaw and banana wine), 18.00 ± 0.02 % (pawpaw, banana and watermelon wine) and 18.50 ± 0.02 % (banana and watermelon wine). This finding agree with the work of Bechem et al. (2007) that palm wine yeast isolates may show a range of 10-20 % alcohol tolerance. Also, Noll (2008) reported that the strains of the yeast (*S.cerevisiae*) isolated from palm wine are different genetically from the yeast strains that are used to make wine from grapes and have the ability to survive and continue fermentation process up to ethanol concentration of 18 %, making them ideal candidate for producing ethanol for fuel.

The performance and potential of the test yeast as substitute for commercial baker’s yeast was measured by the amount of alcohol produced. The alcohol produced by the test yeast in this study were high (16.00–18.50 %) compared to the studies on commercial yeast (10.46 %) as reported by Chilaka et al. (2010). High alcohols are known to be important precursors for the formation of esters, which are associated with pleasant aromas Clement-Jimenez et al. 2005). Reports have shown that alcoholic fermentation leads to a series of by-products in addition to ethanol. Some of the by-products include carbonyl compounds, alcohols, esters, acids and acetyls. All of which influence the quality of the finished product. The composition and concentration levels of the by-products can vary widely (Duarte et al. 2010). In general, the concentrations of ethanol contribute to the whole characteristic quality and flavour of the produced wine (Reddy and Reddy 2009, 2004). However, the amount of alcohol produced by the test yeast were not observed to differ significantly (p > 0.05).

In the present investigation, the test fermentation yeast (*S.cerevisiae*) was the only organism isolated from pawpaw and watermelon wine as well as pawpaw and banana wine while neither pawpaw, banana and watermelon wine nor banana and watermelon wine showed the presence of any microorganism. This is an indication of good quality. This observation may be attributed to low pH values, high acidity and high alcohol contents of the wines which are known to inhibit the growth of pathogens and gives fermenting yeast a competitive advantage in natural environment as reported by Reddy and Reddy (2005) and Chilaka et al. (2010). The absence of the growth of the yeast in pawpaw, banana and watermelon wine and banana and watermelon wine could be due to the high alcoholic content which exceeded the ethanolic tolerance level of the yeast used for fermentation.

The colours of the wines in the present study were observed to be pale yellow (pawpaw and watermelon wine), straw yellow (pawpaw and banana wine), dark brown (pawpaw, banana and watermelon wine) and cream (banana and watermelon wine). This is an indication that the combination of the fruits served as a good substrate for wine production with pawpaw and banana being the most efficient as shown in this study. The good aroma obtained in the wines could be attributed to high alcohol content in accordance with the report of Clement-Jimenez et al. (2005).

Sensory evaluation rated the wines acceptability as pawpaw and banana wine > pawpaw and watermelon > pawpaw, watermelon and banana > banana and watermelon wine. These attributes compared favourably with the reports for other tropical wines (Akubor et al. 2003; Ray et al. 2011; Sahu et al. 2012; Panda et al. 2014a, b). Also, the sensory evaluation of the wines in the present study do not differ significantly (P > 0.05).

## Conclusion

The present study which was based on the evaluation of three indigenous fruits as substrates for wine production and the efficiency of isolated *S.cerevisiae* from palm wine for mixed fruit wine production has revealed that the three test fruits (pawpaw, banana and watermelon) are good substrates for wine production. The biochemical and sensory attributes of the wines were acceptable by the consumers. The study has also given an insight into the efficacy and role of *S. cerevisiae* from palm wine during alcoholic fermentation of fruits. Pawpaw, banana and watermelon have short shelf-life under the prevailing temperature and humidity condition in Nigeria. Therefore, this study provides an avenue to preserve their nutrients, minerals, vitamins, aroma and taste to the consumers by fermenting them into wines.
